# Multiple injuries after earthquakes: a retrospective analysis on 1,871 injured patients from the 2008 Wenchuan earthquake

**DOI:** 10.1186/cc11349

**Published:** 2012-05-17

**Authors:** Zhao Lu-Ping, Jose Manuel Rodriguez-Llanes, Wu Qi, Barbara van den Oever, Lina Westman, Manuel Albela, Pan Liang, Chen Gao, Zhang De-Sheng, Melany Hughes, Johan von Schreeb, Debarati Guha-Sapir

**Affiliations:** 1 People's Hospital of Deyang City, Taishian North Road 173, Deyang city, 618000, China; 2 Centre for Research on the Epidemiology of Disasters, Institute of Health and Society, Université catholique de Louvain, Clos Chapelle-aux-Champs 30, Brussels, 1200, Belgium; 3 Division of Global Health (IHCAR), Karolinska Institute, Nobels väg 9, Stockholm, SE-171 77, Sweden

## Abstract

**Introduction:**

Multiple injuries have been highlighted as an important clinical dimension of the injury profile following earthquakes, but studies are scarce. We investigated the pattern and combination of injuries among patients with two injuries following the 2008 Wenchuan earthquake. We also described the general injury profile, causes of injury and socio-demographic characteristics of the injured patients.

**Methods:**

A retrospective hospital-based analysis of 1,871 earthquake injured patients, totaling 3,177 injuries, admitted between 12 and 31 May 2008 to the People's Hospital of Deyang city (PHDC). An electronic, webserver-based database with International Classification of Diseases (ICD)-10-based classification of earthquake-related injury diagnoses (IDs), anatomical sites and additional background variables of the inpatients was used. We analyzed this dataset for injury profile and number of injuries per patient. We then included all patients (856) with two injuries for more in-depth analysis. Possible spatial anatomical associations were determined *a priori*. Cross-tabulation and more complex frequency matrices for combination analyses were used to investigate the injury profile.

**Results:**

Out of the 1,871 injured patients, 810 (43.3%) presented with a single injury. The rest had multiple injuries; 856 (45.8%) had two, 169 (9.0%) patients had three, 32 (1.7%) presented with four injuries, while only 4 (0.2%) were diagnosed with five injuries. The injury diagnoses of patients presenting with two-injuries showed important anatomical intra-site or neighboring clustering, which explained 49.1% of the combinations. For fractures, the result was even more marked as spatial clustering explained 57.9% of the association pattern. The most frequent combination of IDs was a double-fracture, affecting 20.7% of the two-injury patients (*n *= 177). Another 108 patients (12.6%) presented with fractures associated with crush injury and organ-soft tissue injury. Of the 3,177 injuries, 1,476 (46.5%) were fractures. Most injuries were located in the head (22.9%) and lower extremities (30.8%).

**Conclusions:**

Multiple injuries are put forward as an important component of the injury profile after this earthquake. A pattern of injury combinations and spatial aggregation of injuries was also found. Clinical diagnosis and treatment should be adapted to care of these patients. More studies are needed to generalize these findings.

## Introduction

In the past 20 years, earthquakes have killed 509,229 and injured 1,462,321 people [1]. Adequate and timely management to reduce mortality and morbidity of the injured in earthquakes is of concern to rescuers and medical-care professionals [2,3]. Although many earthquake victims suffer injuries requiring surgical intervention, major gaps in knowledge on the epidemiology of earthquakes compromise the surgical delivery. What is the burden and distribution of surgical conditions after earthquakes? Do they vary depending on context? Research following the 1988 earthquake in Armenia showed that superficial, head and lower extremities injuries accounted for the majority of the cases [4]. This was consistent with the findings of earlier studies [5,6]. A number of later studies emphasized the importance of crush injury and fractures, which often represent more than half of the recorded injuries [7-9].

The first decade of the 21^st ^century will be remembered for extremely deadly earthquakes, such as the 2003 Bam (Iran) earthquake, the 2005 Kashmir (Pakistan) earthquake, the 2008 earthquake in Sichuan (China) or the more recent January 2010 event in Haiti. Most study results from these earthquakes [10-20] were coincident with earlier reports, although their comparison is not always straightforward, mainly due to a lack of standardization of definitions for injury classification. The study of combination injuries has received even less attention. Despite early studies documenting that 39.7 to 51.3% of patients presented with more than one injury [4,6,21], little specific research has been conducted on the profile of multiple injuries after earthquakes.

On the afternoon of 12 May 2008, a 7.9-magnitude (Mw) earthquake hit Sichuan Province, a mountainous region in Western China. The Chinese Ministry of Health reported 68,858 deaths, 18,618 missing and 374,000 injured [22]. Several reports describing the general injury profile have been published since this earthquake [15-19,23]. However, only two of them assessed the injury profile in a large sample of patients treated in a single facility [19,23]. The West China Hospital of Sichuan University at Chengdu city treated 2,728 people injured by the disaster. Of the 1,861 patients studied, 55% had fractures, 10% had craniocerebral injuries, and 7.5% presented with thoracoabdominal injuries [23]. Researchers from the second Military University worked in a local hospital of Jiangyou city. They studied 1,038 patients out of 3,038 treated at the facility. Of these, 40% presented with fractures, and 15% had craniocerebral injuries [19]. In general, reports on multiple injuries after this earthquake have been anecdotal [16,19], or used unclear methods [19].

More and better information about the types and profiles of these multiple injuries are needed. This can add essential information to better plan and more readily adapt the surgical management of the injured following earthquakes. This research aims to investigate the injury profile of the Wenchuan earthquake in patients admitted to the People's Hospital of Deyang City (PHDC), Sichuan province, with special focus on the pattern of multiple injuries.

## Materials and methods

This study was approved by the Ethical Committee of the People's Hospital of Deyang city. No informed consent was necessary as this study used existing data.

This study retrospectively investigated the clinical records of 1,871 patients admitted to the People's Hospital of Deyang city (PHDC) with earthquake-related injuries during the 20 days that followed the 12 May 2008 Wenchuan earthquake. We used descriptive statistics to gain insight into the demographics, cause of injury, transfer of patients and the general injury profile of the injured patients admitted to the PHDC after the earthquake. In patients presenting with two injuries, the pattern of aggregation of these injuries by location and by injury diagnoses were further explored. The association of anatomical sites in patients presenting with two fractures was also studied.

### Study site

The earthquake struck in Wenchuan, a county of the Sichuan province, People's Republic of China. All hospitals not destroyed during the earthquake were part of the relief efforts. The data for this study were collected at the PHDC, the largest Level 3, Grade 1 state-owned hospital in Deyang District and the closest (99 km east) to the epicenter. The catchment area of the hospital covers a population of four million and has a capacity of 1,200 beds. The hospital has 1,462 working staff, of which there are 435 doctors, 743 nurses and 130 paramedical personnel. The PHDC has 30 medical departments, 10 technical sections, 20 inpatient sectors, 1 intensive care unit (ICU) and 4 clinical departments of provincial importance (including orthopedics, general surgery, neurosurgery and neurology). The hospital was partly damaged by the earthquake but none of the diagnostic machines were damaged, and the hospital was able to provide health care free of charge immediately after the earthquake.

### Study design and database development

**Figure 1:**
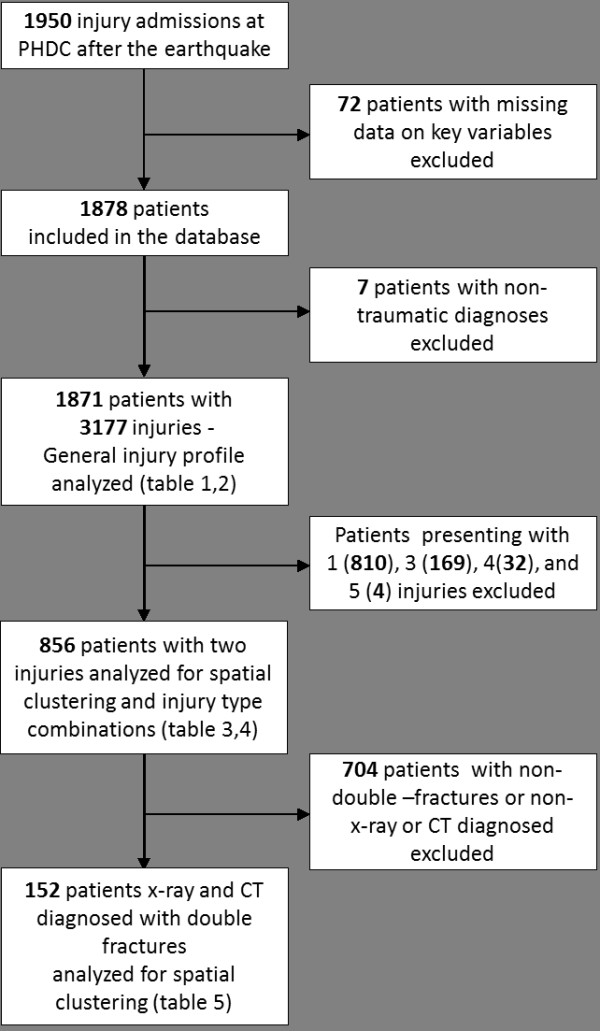
**Flow diagram of the sample obtained at the People's Hospital of Deyang city (PHDC)**.

An electronic, webserver-based database with information on earthquake-related injuries from patients admitted to the People's Hospital of Deyang city between 12 and 31 May 2008 was produced based on patient files available at the hospital. First, an empty patient record was translated from Mandarin into English. The listed variables were discussed with the PHDC's medical experts, and a quality check of the original and translated versions were carried out with the help of a translator. The listed variables were reviewed and inclusion and exclusion criteria were decided upon (Figure 1). A codebook defining the retained variables was jointly developed by PHDC and researchers from the Centre of Research of the Epidemiology of Disasters (CRED). This codebook also assigned clear categories to the data whenever possible, so as to minimize data entry errors. A data entry manual was developed and used to train data entry operators; the quality of the data entry process was checked by CRED researchers. Data entry was performed between 12 March and 5 May 2010. A total of 52 variables were recorded, including data on demographics, admission and discharge information, injury diagnosis and diagnosis accordance, surgical treatment and procedures performed. We recorded a maximum of five injuries per patient as only four patients had five injuries.

### Study variables

This analysis used 10 variables out of the 52 available. The outcome of the study was the injury diagnoses of the patients admitted to the hospital. Injury diagnoses were attributed following the International Classification of Diseases (ICD)-10 (version 2007) [24]. We used the discharge ICD-10 code and injury diagnosis in the analysis. A binary variable indicating whether patients presented with a single or multiple injuries was produced and used to group the patients' injury profile (see Table 1). Study records were classified by body region and injury diagnoses using a modified version of the classification adopted by the ICD-10. Abdomen, lower back, lumbar spine and pelvis - a single category in the ICD-10, were separated into three different anatomical sites: abdomen, lower back - which comprised the lumbar spine, and pelvis. The rest of anatomical sites appearing in ICD-10 remained unchanged. Reported injury diagnoses were based on the same classification with minor adjustments (Table 2). The category named contusion included hematomas of the scalp. The category labeled as laceration encompassed lacerations, bites, cuts, open wounds and minor avulsions.

**Table 1:** Characteristics of singleinjury and multiple-injury patients.

Characteristics		Admitted (*n *= 1,871)	Single injury (*n *= 810)	Multiple injuries (*n *= 1,061)
**Demographics**				
Sex, no.* (%)				
	Female	936 (50.1)	402 (49.6)	534 (50.4)
	Male	934 (49.9)	408 (50.4)	526 (49.6)
Age, no.† (%)				
	≤15	204 (10.9)	64 (7.9)	140 (13.3)
	> 15 to 65	1,305 (69.9)	575 (71.0)	730 (69.1)
	> 65	357 (19.1)	171 (21.1)	186 (17.6)
Marital status, no.‡ (%)				
	Single	54 (3.4)	24 (3.4)	30 (3.5)
	Married	1,506 (95.9)	676 (96.2)	830 (95.7)
	Divorced	1 (0.1)	0 (0.0)	1 (0.1)
	Widowed	9 (0.6)	3 (0.4)	6 (0.7)
Occupation, no.§ (%)				
	Farmer	1016 (54.4)	464 (57.3)	552 (52.1)
	Worker	16 (0.9)	9 (1.1)	7 (0.7)
	Officer	409 (21.9)	161 (19.9)	248 (23.4)
	Student	240 (12.8)	85 (10.5)	155 (14.6)
	Other	188 (10.1)	91 (11.2)	97 (9.2)
**Cause of injury**				
Due to main earthquake or aftershock?¶				
	Main shock	1,655 (88.7)	689 (85.6)	966 (91.1)
	Aftershock	210 (11.3)	116 (14.4)	94 (8.9)
Directly or indirectly caused by the earthquake?**				
	Direct	1,844 (99.4)	796 (98.9)	1,048 (99.8)
	Indirect	11 (0.6)	9 (1.1)	2 (0.2)
**Transfer of patients**				
Was the patient transferred to another hospital?				
	Yes	320 (17.1)	99 (12.2)	221 (20.8)
	No	1,551 (82.9)	711 (87.8)	840 (79.2)

*One observation with missing data in the multiple-injuries group. †Five observations with missing data in the multiple-injuries group. ‡Includes only those patients in marriage age - 22 years-old in males, 20 years-old in females. Nine observations with missing data, one in the single-injury group, and eight in the multiple-injuries group. §Two observations with missing data in the multiple-injuries group. ¶Six observations with missing data, five in the single-injury group, and one in the multiple-injuries group. ** Sixteen observations with missing data, five in the single-injury group and eleven in the multiple-injuries group

**Table 2:** Diagnoses by body region of 3,177 injury diagnoses registered at People's Hospital of Deyang City after the 2008 Sichuan earthquake, China

Body region	Head	Neck	Thorax	Abdomen	Lower back	Pelvis	Shoulder, upper arm	Elbow, forearm	Wrist, hand	Hip, thigh	Knee, lower leg	Ankle, foot	NOS	Total, no. (%)
**Injury diagnoses**														
Contusion	363*	0	62	3	0	0	1	0	26	20	22	9	0	506 (15.9)
Superficial injury NOS	0	0	0	0	0	0	0	0	0	11	2	0	265	278 (8.8)
Laceration†	42	0	0	9	0	0	14	3	7	16	145	35	0	271 (8.5)
Fracture	109	9	228	0	130	116	136	82	56	146	327	137	0	1476 (46.5)
Dislocation	0	0	0	0	0	1	8	1	2	14	14	9	0	49 (1.5)
Sprain	0	0	0	0	0	0	0	0	0	0	3	1	0	4 (0.1)
Injury of organs, soft tissues and NOS	200‡	1	122§	105	3	0	1	1	0	1	1	0	0	435 (13.7)
Crush injury	13	0	19	18	18	3	1	1	0	0	20	19	0	112 (3.5)
Amputation	0	0	0	0	0	0	0	1	7	0	26	3	0	37 (1.2)
Burns (second degree)	0	0	2	0	0	0	0	0	0	0	0	0	0	2 (0.1)
Compartment syndrome	0	0	0	0	0	0	0	0	0	0	0	0	7	7 (0.2)
**Total, no. (%)**	727 (22.9)	10 (0.3)	433 (13.6)	135 (4.2)	151 (4.8)	120 (3.8)	161 (5.1)	89 (2.8)	98 (3.1)	208 (6.5)	560 (17.6)	213 (6.7)	272 (8.6)	**3177**

*Includes hematoma of scalp (*n *= 61). †This includes bites, cuts, open wounds and minor avulsions. ‡Includes intracranial bleeds (*n *= 105) and traumatic brain injuries (*n *= 92). §Including pleural collections (*n *= 53) - consisting of pleural effusion, hemothorax and hydrothorax, pneumothoraces (*n *= 5) and pleural collections with pneumothorax (*n *= 15). NOS, not otherwise specified.

The demographic variables considered were patients' sex, age, marital status and occupation (Table 1). Age was a continuous variable in the original database, but was reclassified into three broad groups (in years): ≤15, > 15 to 65, > 65, to facilitate its visualization and comparison with Sichuan census data. Marital status included four categories: single, married, divorced and widowed. Data were only shown for those patients of marriage age: 22 years-old in males and 20 years-old in females (Table 1). Occupation was categorized in broad classes widely used in the Chinese setting: farmers, workers, officers, students and others. Two variables reported on the patient's cause of injury. First, patients were asked whether they were injured during the main shock or during subsequent aftershocks. Second, patients were queried to determine if the injury was caused directly by the shaking or by indirect causes. Direct causes included falling objects, collapsed building, car crashes at the time of the earthquake, fire or electroshock. Indirect causes were related to rescue efforts, car crashes after the earthquake and injuries due to damaged infrastructure. Transferred patients were also recorded and coded as a dichotomized variable. A variable reported on the diagnostic tool used to determine each injury, and included five categories: computed tomography (CT), x-rays, color Doppler, physical examination and lab test. It was used to select only those fractures diagnosed using CT and x-rays.

Spatial clustering of injuries and associated injury types were studied for patients presenting with two injuries. These patients represented nearly half of the case load. For the spatial analysis of combined injuries, we identified injuries occurring within the same body region and those in neighboring anatomical sites. The first group corresponded to the 12 body regions used throughout the study: head, neck, thorax, shoulder-upper arm, elbow-forearm, wrist-hand, abdomen, lower back, pelvis, hip-thigh, knee-lower leg, and ankle-foot. Adjacent body regions accounted for 13 other possibilities: head-neck, neck-thorax, thorax-lower back, thorax-abdomen, thorax-shoulder-upper arm, shoulder-upper arm-elbow-forearm, elbow-forearm-wrist-hand, abdomen-pelvis, abdomen-lower back, pelvis-lower back, pelvis-hip-thigh, hip-thigh-knee-lower leg, and knee-lower leg-ankle-foot. The not otherwise specified, NOS, category indicated injuries difficult to define anatomically. Most of these corresponded to superficial injuries. Overall, 78 site combinations were possible with 25 relevant to understand the spatial clustering of injury associations.

### Sample selection and data analysis

A total of 1,950 patients with earthquake-related injuries were admitted to the hospital. Of these, 72 patients were excluded due to missing data for key variables (Figure 1). Patient data were entered for the remaining 1,878 patients. Seven patients with non-traumatic diagnoses were excluded from further analysis (Figure 1). The leftover 1,871 patients, which accounted for 3,177 injury diagnoses, were used in the analysis of the general injury profile (Tables 1 and 2). Of these patients, 810 (43.3%) presented with a single injury, 856 (45.8%) with two, 169 (9.0%) patients were diagnosed with three, 32 (1.7%) presented four injury diagnoses, and only 4 (0.2%) injured patients were diagnosed with five injuries (Figure 1). More in-depth analyses were undertaken for the 856 patients presenting with two injuries (Figure 1, Tables 3 and 4). One hundred seventy-seven patients out of these 856 were diagnosed with two fractures. Of these, only 152 patients with both injuries diagnosed either through CT or x-rays were included in more specific analysis (Figure 1 and Table 5). Tables 3 to 5 reported on associations contributing at least one percent of the cases. Additional files show full matrices with combination frequencies and associated percentages (Additional files 1, 2, 3).

**Table 3:** Most frequent combinations of injury diagnoses by body region in 856 two-injury patients.

Associated body regions	n (%)
Single body region	
Head	112 (13.1)
Thorax	61 (7.1)
Knee-lower leg	45 (5.3)
Ankle-foot	27 (3.2)
Abdomen	14 (1.6)
Pelvis	9 (1.1)
Multiple body regions	
Head, knee-lower leg	52 (6.1)
Head, NOS	44 (5.1)
Knee-lower leg, ankle-foot†	27 (3.2)
Knee-lower leg, NOS	24 (2.8)
Head, thorax	23 (2.7)
Hip-thigh, knee-lower leg†	23 (2.7)
Lower back, knee-lower leg	23 (2.7)
Thorax, knee-lower leg	22 (2.6)
Thorax, lower back†	20 (2.3)
Thorax, NOS	20 (2.3)
Head, lower back	17 (2.0)
Shoulder-upper arm, knee-lower leg	17 (2.0)
Head, hip-thigh	12 (1.4)
Head, pelvis	9 (1.1)
Thorax, shoulder-upper arm†	9 (1.1)
Shoulder-upper arm, NOS	9 (1.1)

* Includes injury combinations accounting for more than one percent of the sample. † Indicates neighboring body regions. IDs, injury diagnoses; PHDC, People's Hospital of Deyang city; NOS, not otherwise specified.

**Table 4:** Most frequent combinations of injury diagnosess in 856 two-injury patients.

Associated injury diagnoses	n (%)
Similar injury diagnoses	
Fracture	177 (20.7)
Contusion	24 (2.8)
Lacerations	25 (2.9)
Two injury diagnoses	
Fracture, contusion	107 (12.5)
Fracture, laceration	83 (9.7)
Fracture, superficial injury NOS	53 (6.2)
Fracture, injury NOS	38 (4.4)
Contusion, superficial injury NOS	36 (4.2)
Fracture, crush injury	29 (3.4)
Laceration, superficial injury NOS	20 (2.3)
Laceration, contusion	19 (2.2)
Fracture, pleural collection	15 (1.8)
Fracture, intracranial bleeding	13 (1.5)
Fracture, hematoma	13 (1.5)

* Includes injury combinations accounting for more than one percent of the sample. IDs, injury diagnoses; PHDC, People's Hospital of Deyang city; NOS, not otherwise specified.

**Table 5:** Most frequent combinations of fractures by body region in 152 patients with two-fractures.

Body regions	n (%)
Single body region	
Ankle-foot	14 (9.2)
Elbow, forearm	7 (4.6)
Pelvis	6 (3.9)
Knee-lower leg	4 (2.6)
Thorax	4 (2.6)
Hip, thigh	3 (2.0)
Shoulder, upper arm	3 (2.0)
Multiple body regions	
Thorax, lower back†	14 (9.2)
Knee-lower leg, ankle-foot†	8 (5.3)
Pelvis, hip-thigh†	6 (3.9)
Thorax, elbow-forearm	5 (3.3)
Shoulder-upper arm, wrist-hand	5 (3.3)
Elbow-forearm, wrist-hand†	5 (3.3)
Hip-thigh, knee-lower leg†	5 (3.3)
Thorax, knee-lower leg	5 (3.3)
Lower back, ankle-foot	5 (3.3)
Shoulder-upper arm, knee-lower leg	5 (3.3)
Thorax, shoulder-upper arm†	4 (2.6)
Lower back, knee-lower leg	4 (2.6)
Shoulder-upper arm, ankle-foot	4 (2.6)
Lower back, pelvis†	3 (2.0)
Thorax, ankle-foot	3 (2.0)
Pelvis, elbow-forearm	3 (2.0)
Elbow-forearm, knee-lower leg	3 (2.0)
Head, knee-lower leg	2 (1.3)
Lower back, elbow-forearm	2 (1.3)
Hip-thigh, ankle-foot	2 (1.3)

* Includes injury combinations accounting for more than one percent of the sample. Only 152 patients for whom both fractures were diagnosed using X-rays or computed tomography were included. Twenty-five patients were excluded. † Indicates neighboring body regions. IDs, injury diagnoses; NOS, not otherwise specified; PHDC, People's Hospital of Deyang city.

Descriptive statistics were used throughout the study. We did not make use of association measures, such as relative risk, odds ratio or correlation coefficients, due to the inability to clearly define the population generating the data.

## Results

### Demographics

Table 1 lists the characteristics of the injured patients admitted to PHDC, and those presenting with single and multiple injuries. The proportion of injured females admitted to the hospital was slightly higher than that of males (50.1 and 49.9%, respectively). More males than females presented with single-injuries (50.4%). Contrarily, more females with multiple-injuries were admitted to the hospital (50.5%). A larger proportion of patients with multiple injuries were observed in the under-16 group relative to the other age groups (see Table 1).

More than half of the patients admitted to PHDC were farmers (54.4%). Relative to other occupation categories, farmers presented slightly more often with a single-injury (57.3%) and less often with more than one injury (52.1%). On the other side, officers and students presented more often with multiple-injuries (23.4% and 14.6%, respectively) than did with single-injuries (19.9% and 10.5%).

### Cause of injury and transfer of patients

More than 1/10 of the patients analyzed were injured during aftershocks (Table 1). These patients presented more often with single-injuries (14.4%) than with multiple-injuries (8.9%). Most injuries of the patients admitted to the hospital were a direct consequence of the earthquake (99.4%). A significant proportion of patients were transferred to tertiary referral hospitals (17.1%). Transferred patients were more frequently those presenting with multiple injuries (20.8% vs. 12.2%).

### General injury profile

A total of 3,177 injury diagnoses were recorded in 1,871 patients (Table 2). A large share of these injuries were fractures (46.5%), followed by contusions (15.9%), injuries of organs and soft tissues (13.7%), superficial injuries (8.8%) and lacerations, bites, cuts, open wounds and minor avulsions (8.5%). A total of 112 crush injuries (3.5%) were diagnosed in 111 patients. Dislocations and sprains were rare (1.5% and 0.1%, respectively). Thirty-seven traumatic amputations occurred (1.2%). Burn injuries and compartment syndrome were not frequent in the sample, accounting for 0.1% and 0.2%, respectively (see Table 2). By body region, head (22.9%) and lower extremities (that is, upper leg, lower leg and ankle-foot) took a large part of the injury burden (30.8%; see Table 2).

Table 2 also presents the most relevant injury diagnoses by body region. Contusion, which includes scalp hematomas, was the most frequent head-injury diagnosis (49.9%), followed by soft tissue and organ injury (27.5%) and fractures (15%). Neck fracture was the most important diagnosed injury at this anatomical site (90%). Fractures were the predominant diagnosis in most remaining body regions: thorax (52.7%), lower back (86.1%), pelvis (96.7%), shoulder-upper arm (84.5%), elbow-forearm (92.1%), wrist-hand (57.1%), hip-thigh (70.2%), knee-lower leg (58.4%), and ankle-foot (64.3%). Logically, this was not the case at the abdomen, in which organ and soft tissue injury were very common (77.8%). The abdominal region likewise provided an important contribution to overall crush injuries (16.1%).

The data presented in Table 2 also show the distribution of each injury diagnosis across body regions. The largest proportions of fractures occurred in the knee-lower leg (22.2%) and thorax (15.4%). Most contusions were identified at the head (71.7%). Lacerations were mostly diagnosed in the knee-lower leg (53.5%). Seventy-two percent of all lacerations were located on the lower extremities. Organ and soft tissue injuries were mostly located in the head (46.0%), thorax (28.0%) and abdomen (24.1%). Crush injuries were especially frequent in the trunk, head and lower leg. Most amputations were performed at the lower-leg level (70.3%) and all burn injuries involved the thorax. Dislocation and sprain were rare but clustered in the lower extremities (75.6% and 100%, respectively).

### Pattern of multiple injuries in patients with two IDs

#### Body region

A two-level spatial clustering was detected among the 856 patients presenting with two IDs (Table 3). First, more than one-third of these patients (35%) presented both IDs within the same body region. Six of the 12 body regions took 31.4% of this share: head (13.1%), thorax (7.1%), knee-lower leg (5.3%), ankle-foot (3.2%), abdomen (1.6%) and pelvis (1.1%).

A second level of spatial aggregation appeared in neighboring anatomical regions, such as the thorax and lower back (2.7%) or thorax-shoulder-upper arm (1.1%). This spatial pattern appeared specially clustered in the lower limbs: lower leg and ankle-foot (3.2%) and upper leg-lower leg (2.7%). The 10 former figures explained more than 40% of the cases. A remarkable exception was the high frequency of IDs combinations among knee-lower leg and head (6.1%).

#### Injury diagnoses

IDs were also clustered in patients diagnosed with two injuries. A patient presenting with two fractures was the prominent profile of this group (20.7%; Table 4). More than 1/10 of the patients with two IDs (12.5%) presented a contusion combined with a fracture, about 10% (9.7%) a laceration with a fracture or a fracture with other superficial non-specified injury (6.2%). All the previous accounted for almost half the combinations observed. Fractures were importantly detected accompanying soft tissue and organ injuries, mostly located in thorax and abdomen (4.4%), traumatic brain injuries and intracranial bleeding (3.1%), and pleural collections (1.8%). Fractures were also found to be associated with crush injuries (3.4%). Superficial injuries, such as contusions, lacerations and unspecified superficial injuries were also frequently presented in association (see Table 4).

#### Anatomical sites associated in patients with two fractures

A two-level clustering pattern, similar to that observed for all IDs across body regions, was observed for this subset of 152 patients. A spatial clustering within body regions was detected. In this case, ankle-foot (9.2%), elbow-forearm (4.6%) and pelvis (3.9%) accounted for most of these intra-site associations. Knee-lower leg (2.6%), thorax (2.6%), hip-thigh (2.0%), and shoulder-upper arm, followed in importance (Table 5). In general, intra-site associations did account for less than the 35% observed considering all IDs (27.6%), and the seven previous sites accounted for most of this pattern (26.9%). In contrast, fractures in neighboring anatomical sites gained importance when only fractures were considered. Overall, nearly 33% of the patients (32.9%) presenting with two fractures had those in neighboring body regions and seven combinations explained most of this percentage (29.6%). Thorax and lower back (9.2%), knee-lower leg and ankle-foot (5.3%), pelvis and hip-thigh (3.9%) accounted for most of these associations (Table 5).

## Discussion

Our study showed that more than half of the injured arriving at a hospital close to the epicenter of a major earthquake had multiple injuries. Almost half of those with two injuries had injuries in the same or in a neighboring body part. The most frequent combination injury was two fractures. These findings are important to plan and adapt surgical management of injury following earthquakes. It is noteworthy that only four patients presented with five injuries. It could be that those with more injuries died due to the severity of multiple injuries. The fact that 45% of all injuries were fractures corresponds well with previous earthquake studies [10,14]. It also corresponds well with the frequencies of injuries observed in some Wenchuan earthquake studies that reported injury profiles [19,23]. However, we have found no comparable study on multiple injury profiles related to this earthquake. Our study reviewed data collected on patients arriving 10 minutes after the earthquake, differentiating it from the injury profile studies completed by the foreign field hospitals that arrived several days after the earthquake. This fact probably impacted on the injury profile and severity of the injuries observed in our study.

### Demographics

The proportion of patients younger than 15 years of age (10.9%) was underrepresented when compared with Chinese National Census data for that age group (17.4%) [25]. In contrast, patients older than 65 (19.1%) were over-represented compared with the same census (11.4%). Many explanations are possible for these figures, including differential exposure, behavior, injury severity or mortality, varying degrees of access to the hospital as well as seasonal work-related population movements.

Interestingly, a higher percentage of farmers presented with single injuries (57.3%) rather than with multiple injuries (52.1%), while the results were reversed for officers and students. This might be influenced by the fact that officers and students were in larger buildings. It is, however, impossible to ascertain the reasons behind the above figures without in-depth population-based surveys of the exposed populations.

### Cause of injury and transfer of patients

Over 1/10 of the inpatients were injured in aftershocks. Even though large-magnitude earthquakes often lead to subsequent damaging earthquakes [26], little attention has been paid to the injury burden produced by aftershocks. In this study, less than 1% of the patients were injured by indirect causes, which supports results from previous studies [6].

### Clinical significance of the multiple injury profile

The results of this study provide important information for the clinical management of the earthquake-injured. Surgical teams arriving at the site must be prepared to care for patients with multiple injuries. This requires timely and adaptable care in patients who may be at increased risk of sepsis and multi-organ failure. Not only are the hospitals' capacities often overwhelmed by the massive and unexpected load of patients, but the patients must be triaged and treated in an austere environment where delayed referral is a common phenomenon. In addition, the hospital has to care for the normal caseload of patients that arrive on a daily basis. Assuming that multiple-injured patients are a frequent phenomenon after earthquakes, trauma care after earthquakes will necessitate an increased need for logistical and human resource capacities (for example, essential surgical supplies and experienced surgeons). It also helps to more clearly define the need for triage-based surgical strategies to optimally use the limited available resources. Such theories have found support in the evaluation of medical assistance given after the earthquake in Haiti 2010 [27].

The principles of fracture management in polytrauma patients have been a controversial area of research for some years. In the 1980s, the concept of early total care (ETC) was developed after studies reporting a drastically reduced complication rate if femur fractures were stabilized immediately. However, application of ETC has been reported as non-beneficial in polytrauma patients [28]. In such cases, the concept of initial temporary fixation and secondary conversion to a definitive procedure has been advocated. This approach is often referred to as "damage control orthopaedics." In situations of delayed referral, which often occur in rural areas, damage control provides an acceptable method of treatment in the management of polytrauma cases [29]. The approach used will depend on available resources as well as the number of patients and the severity of injuries.

### Limitations

This study retrospectively analyzed patient files registered by many different doctors. It is impossible to verify the intra-doctor and inter-doctor variability in recording the correct diagnosis. To balance this, we systematically used the discharge diagnosis, and when available we used objective x-ray or CT verification of fractures. In addition, the hospital has been using ICD-10 for injury classification since 2003 for which all doctors were asked to undergo training. The systematic use of this classification system very likely contributed to reduce this variability in the study. Another limitation of the study is that its facility-based design does not allow extrapolations of the results to the general population affected by the earthquake. To date, there has been no comprehensive population-based injury study from earthquakes.

## Conclusions

Our study suggests that multiple injuries are an important component of the injury profile after the 2008 Wenchuan earthquake. The study found that two-injury combinations are spatially clustered in the same or neighboring anatomical sites and this effect is more evident when only fractures are considered. These findings have implications for surgical preparedness to earthquakes and the clinical management of the injured.

## Key messages

• Multiple injuries are likely an important feature of the earthquake injury profile.

• The surgical response to earthquakes must be prepared to care for patients with multiple injuries.

## Abbreviations

CRED: Centre for Research on the Epidemiology of Disasters; CT: computed tomography; ETC: early total care; ICD: International Classification of Diseases; ICU: intensive care unit; IDs: injury diagnoses; Mw: mechanical work - symbol for the moment magnitude scale of an earthquake; PHDC: People's Hospital of Deyang city.

## Competing interests

The authors declare that they have no competing interests.

## Authors' contributions

DG-S obtained the funding. BvdO, MA, ZL-P, WQ, CG and DG-S designed and implemented the database system. ZL-P, WQ, PL, CG and ZD-S collected the data. JMR-L, BvdO, MA, ZL-P, WQ and PL cleaned the data. JMR-L, BvdO, JvS, LW, MH and DG-S conceived the idea of the paper. JMR-L designed the study, analyzed the data and drafted the manuscript. JvS, MH, LW, MA, BvdO and DG-S contributed to study design and drafting of the manuscript. MA provided technical support. All authors critically revised the manuscript for important intellectual content. All authors read and approved the final version of the manuscript.

## Supplementary Material

Additional file 1 **Frequency (%) matrix of the combinations of IDs by body region for the 856 patients admitted with two IDs to PHDC, Sichuan province, China**. Click here for file

Additional file 2 **Frequency (%) matrix of combinations of IDs for the 856 patients admitted with two IDs to PHDC, Sichuan province, China**. Click here for file

Additional file 3 Frequency (%) matrix of fractures by body region for the 152 patients admitted with two fractures to PHDC, Sichuan province, China*. Click here for file
