# Supplementary material for: Multiple injuries after earthquakes: a retrospective analysis on 1,871 injured patients from the 2008 Wenchuan earthquake
Source: Crit Care. 2012 May 17;16(3):R87. doi: 10.1186/cc11349 (PMC3580632; doi:10.1186/cc11349)
Supplement: Additional file 1 — Frequency (%) matrix of the combinations of IDs by body region for the 856 patients admitted with two IDs to PHDC, Sichuan province, China. [file cc11349-S1.DOC]

|  | **Head** | **Neck** | **Thorax** | **Abdomen** | **Lower back** | **Pelvis** | **Shoulder,**  **upper arm** | **Elbow, forearm** | **Wrist,**  **hand** | **Hip, thigh** | **Knee,**  **lower leg** | **Ankle,**  **foot** | **NOS** |
| --- | --- | --- | --- | --- | --- | --- | --- | --- | --- | --- | --- | --- | --- |
| **Head** | **112 (13.1)** |  |  |  |  |  |  |  |  |  |  |  |  |
| **Neck** | **2 (0.2)** | **0 (0.0)** |  |  |  |  |  |  |  |  |  |  |  |
| **Thorax** | 23 (2.7) | **1 (0.1)** | **61 (7.1)** |  |  |  |  |  |  |  |  |  |  |
| **Abdomen** | 3 (0.4) | 0 (0.0) | **8 (0.9)** | **14 (1.6)** |  |  |  |  |  |  |  |  |  |
| **Lower back** | 17 (2.0) | 0 (0.0) | **20 (2.3)** | **5 (0.6)** | **1 (0.1)** |  |  |  |  |  |  |  |  |
| **Pelvis** | 9 (1.1) | 0 (0.0) | 7 (0.8) | **7 (0.8)** | **3 (0.4)** | **9 (1.1)** |  |  |  |  |  |  |  |
| **Shoulder, upper arm** | 8 (0.9) | 1 (0.1) | **9 (1.1)** | 1 (0.1) | 1 (0.1) | 1 (0.1) | **8 (0.9)** |  |  |  |  |  |  |
| **Elbow, forearm** | 7 (0.8) | 1 (0.1) | 6 (0.7) | 1 (0.1) | 4 (0.5) | 3 (0.4) | **3 (0.4)** | **7 (0.8)** |  |  |  |  |  |
| **Wrist, hand** | 6 (0.7) | 0 (0.0) | 4 (0.5) | 1 (0.1) | 2 (0.2) | 0 (0.0) | 7 (0.8) | **5 (0.6)** | **8 (0.9)** |  |  |  |  |
| **Hip, thigh** | 12 (1.4) | 0 (0.0) | 0 (0.0) | 5 (0.6) | 0 (0.0) | **7 (0.8)** | 5 (0.6) | 4 (0.5) | 4 (0.5) | **8 (0.9)** |  |  |  |
| **Knee, lower leg** | 52 (6.1) | 1 (0.1) | 22 (2.6) | 2 (0.2) | 23 (2.7) | 1 (0.1) | 17 (2.0) | 8 (0.9) | 3 (0.4) | **23 (2.7)** | **45 (5.3)** |  |  |
| **Ankle, foot** | 8 (0.9) | 0 (0.0) | 5 (0.6) | 5 (0.6) | 6 (0.7) | 1 (0.1) | 5 (0.6) | 0 (0.0) | 2 (0.2) | 5 (0.6) | **27 (3.2)** | **27 (3.2)** |  |
| **NOS** | 44 (5.1) | 0 (0.0) | 20 (2.3) | 5 (0.6) | 4 (0.5) | 4 (0.5) | 9 (1.1) | 0 (0.0) | 2 (0.2) | 7 (0.8) | 24 (2.8) | 8 (0.9) | **0 (0.0)** |
| IDs, injury diagnoses; PHDC, People's Hospital of Deyang city; NOS, not otherwise specified. The diagonal (bold) of the matrix shows intra-site associations. The remaining bold cells indicate neighboring anatomical sites. | | | | | | | | | | | | | |
