# Supplementary material for: Multiple injuries after earthquakes: a retrospective analysis on 1,871 injured patients from the 2008 Wenchuan earthquake
Source: Crit Care. 2012 May 17;16(3):R87. doi: 10.1186/cc11349 (PMC3580632; doi:10.1186/cc11349)
Supplement: Additional file 2 — Frequency (%) matrix of combinations of IDs for the 856 patients admitted with two IDs to PHDC, Sichuan province, China. [file cc11349-S2.DOC]

|  | **A** | **CS** | **C** | **CI** | **D** | **F** | **H** | **INOS** | **IB** | **L** | **PC** | **PNT** | **PNT-PC** | **S** | **SNOS** | **TBI** |
| --- | --- | --- | --- | --- | --- | --- | --- | --- | --- | --- | --- | --- | --- | --- | --- | --- |
| **A** | 0 (0.0) |  |  |  |  |  |  |  |  |  |  |  |  |  |  |  |
| **CS** | 0 (0.0) | 0 (0.0) |  |  |  |  |  |  |  |  |  |  |  |  |  |  |
| **C** | 2 (0.2) | 0 (0.0) | 24 (2.8) | |  |  |  |  |  |  |  |  |  |  |  |  |
| **CI** | 1 (0.1) | 0 (0.0) | 7 (0.8) | 1 (0.1) |  |  |  |  |  |  |  |  |  |  |  |  |
| **D** | 0 (0.0) | 0 (0.0) | 3 (0.4) | 1 (0.1) | 0 (0.0) |  |  |  |  |  |  |  |  |  |  |  |
| **F** | 3 (0.4) | 0 (0.0) | 107 (12.5) | 29 (3.4) | 9 (1.1) | 177 (20.7) |  |  |  |  |  |  |  |  |  |  |
| **H** | 0 (0.0) | 0 (0.0) | 10 (1.2) | 0 (0.0) | 0 (0.0) | 13 (1.5) | 0 (0.0) |  |  |  |  |  |  |  |  |  |
| **INOS** | 1 (0.1) | 1 (0.1) | 6 (0.7) | 4 (0.5) | 0 (0.0) | 38 (4.4) | 3 (0.4) | 7 (0.8) |  |  |  |  |  |  |  |  |
| **IB** | 0 (0.0) | 0 (0.0) | 10 (1.2) | 6 (0.7) | 0 (0.0) | 13 (1.5) | 0 (0.0) | 0 (0.0) | 1 (0.1) |  |  |  |  |  |  |  |
| **L** | 5 (0.6) | 0 (0.0) | 19 (2.2) | 8 (0.9) | 8 (0.9) | 83 (9.7) | 0 (0.0) | 3 (0.4) | 4 (0.5) | 25 (2.9) |  |  |  |  |  |  |
| **PC** | 0 (0.0) | 0 (0.0) | 4 (0.5) | 3 (0.4) | 0 (0.0) | 15 (1.8) | 0 (0.0) | 2 (0.2) | 0 (0.0) | 0 (0.0) | 0 (0.0) |  |  |  |  |  |
| **PNT** | 0 (0.0) | 0 (0.0) | 0 (0.0) | 0 (0.0) | 0 (0.0) | 3 (0.4) | 0 (0.0) | 0 (0.0) | 0 (0.0) | 0 (0.0) | 0 (0.0) | 0 (0.0) |  |  |  |  |
| **PNT-PC** | 0 (0.0) | 0 (0.0) | 2 (0.2) | 3 (0.4) | 0 (0.0) | 6 (0.7) | 0 (0.0) | 0 (0.0) | 0 (0.0) | 0 (0.0) | 0 (0.0) | 0 (0.0) | 0 (0.0) |  |  |  |
| **S** | 0 (0.0) | 0 (0.0) | 1 (0.1) | 0 (0.0) | 0 (0.0) | 0 (0.0) | 0 (0.0) | 0 (0.0) | 0 (0.0) | 1 (0.1) | 0 (0.0) | 0 (0.0) | 0 (0.0) | 0 (0.0) |  |  |
| **SNOS** | 1 (0.1) | 0 (0.0) | 36 (4.2) | 5 (0.6) | 6 (0.7) | 53 (6.2) | 8 (0.9) | 3 (0.4) | 2 (0.2) | 20 (2.3) | 1 (0.1) | 0 (0.0) | 0 (0.0) | 0 (0.0) | 1 (0.1) |  |
| **TBI** | 1 (0.1) | 0 (0.0) | 11 (1.3) | 2 (0.2) | 0 (0.0) | 14 (1.6) | 6 (0.7) | 0 (0.0) | 6 (0.7) | 6 (0.7) | 0 (0.0) | 0 (0.0) | 0 (0.0) | 0 (0.0) | 1 (0.1) | 1 (0.1) |
| IDs, injury diagnoses; PHDC, People's Hospital of Deyang city; NOS, not otherwise specified; A, amputation; CS, compartment syndrome; C, contusion; CI, crush injury; D, dislocation; F, fracture; H, hematoma; INOS, injury NOS; IB, intracranial bleeding; L, laceration; PC, pleural collection; PNT, pneumothorax; PNT-PC, pleural collection with pneumothorax; S, sprain; SNOS, superficial injury NOS; TBI, traumatic brain injury. | | | | | | | | | | | | | | | | |
