# Supplementary material for: Multiple injuries after earthquakes: a retrospective analysis on 1,871 injured patients from the 2008 Wenchuan earthquake
Source: Crit Care. 2012 May 17;16(3):R87. doi: 10.1186/cc11349 (PMC3580632; doi:10.1186/cc11349)
Supplement: Additional file 3 — Frequency (%) matrix of fractures by body region for the 152 patients admitted with two fractures to PHDC, Sichuan province, China*. [file cc11349-S3.DOC]

|  | **Head** | **Neck** | **Thorax** | **Abdomen** | **Lower back** | **Pelvis** | **Shoulder, upper arm** | **Elbow,**  **forearm** | **Wrist, hand** | **Hip, thigh** | **Knee, lower leg** | **Ankle, foot** |
| --- | --- | --- | --- | --- | --- | --- | --- | --- | --- | --- | --- | --- |
| **Head** | **0 (0.0)** |  |  |  |  |  |  |  |  |  |  |  |
| **Neck** | **0 (0.0)** | **0 (0.0)** |  |  |  |  |  |  |  |  |  |  |
| **Thorax** | 0 (0.0) | **0 (0.0)** | **4 (2.6)** |  |  |  |  |  |  |  |  |  |
| **Abdomen** | 0 (0.0) | 0 (0.0) | **0 (0.0)** | **0 (0.0)** |  |  |  |  |  |  |  |  |
| **Lower back** | 1 (0.7) | 0 (0.0) | **14 (9.2)** | **0 (0.0)** | **0 (0.0)** |  |  |  |  |  |  |  |
| **Pelvis** | 0 (0.0) | 0 (0.0) | 3 (2.0) | **0 (0.0)** | **3 (2.0)** | **6 (3.9)** |  |  |  |  |  |  |
| **Shoulder,**  **upper arm** | 1 (0.7) | 1 (0.7) | **4 (2.6)** | 0 (0.0) | 1 (0.7) | 1 (0.7) | **3 (2.0)** |  |  |  |  |  |
| **Elbow,**  **forearm** | 0 (0.0) | 1 (0.7) | 5 (3.3) | 0 (0.0) | 2 (1.3) | 3 (2.0) | **1 (0.7)** | **7 (4.6)** |  |  |  |  |
| **Wrist, hand** | 0 (0.0) | 0 (0.0) | 0 (0.0) | 0 (0.0) | 1 (0.7) | 0 (0.0) | 5 (3.3) | **5 (3.3)** | **1 (0.7)** |  |  |  |
| **Hip, thigh** | 0 (0.0) | 0 (0.0) | 0 (0.0) | 0 (0.0) | 0 (0.0) | **6 (3.9)** | 0 (0.0) | 1 (0.7) | 1 (0.7) | **3 (2.0)** |  |  |
| **Knee,**  **lower leg** | 2 (1.3) | 0 (0.0) | 5 (3.3) | 0 (0.0) | 4 (2.6) | 1 (0.7) | 5 (3.3) | 3 (2.0) | 1 (0.7) | **5 (3.3)** | **4 (2.6)** |  |
| **Ankle, foot** | 0 (0.0) | 0 (0.0) | 3 (2.0) | 0 (0.0) | 5 (3.3) | 1 (0.7) | 4 (2.6) | 0 (0.0) | 1 (0.7) | 2 (1.3) | **8 (5.3)** | **14 (9.2)** |
| *Only 152 patients for whom both fractures were diagnosed using x-rays or computed tomography were included. Twenty five patients were excluded. The diagonal (bold) of the matrix shows intra-site associations. The remaining bold cells indicate neighboring anatomical sites. | | | | | | | | | | | | |
